# Carbogen breathing increases prostate cancer oxygenation: a translational MRI study in murine xenografts and humans

**DOI:** 10.1038/sj.bjc.6604903

**Published:** 2009-02-03

**Authors:** R Alonzi, A R Padhani, R J Maxwell, N J Taylor, J J Stirling, J I Wilson, J A d′Arcy, D J Collins, M I Saunders, P J Hoskin

**Affiliations:** 1 Marie Curie Research Wing, Mount Vernon Hospital, Northwood HA6 2RN, UK; 2 Paul Strickland Scanner Centre, Mount Vernon Hospital, Northwood HA6 2RN, UK; 3 Northern Institute for Cancer Research, Paul O’Gorman Building, Medical School, Framlington Place, Newcastle upon Tyne NE2 4HH, UK; 4 CRUK Clinical MR Research Group, Royal Marsden NHS Foundation Trust, Sutton, Surrey SM2 5PT, UK

**Keywords:** prostate cancer, carbogen, hypoxia, magnetic resonance imaging, BOLD

## Abstract

Hypoxia has been associated with poor local tumour control and relapse in many cancer sites, including carcinoma of the prostate. This translational study tests whether breathing carbogen gas improves the oxygenation of human prostate carcinoma xenografts in mice and in human patients with prostate cancer. A total of 23 DU145 tumour-bearing mice, 17 PC3 tumour-bearing mice and 17 human patients with prostate cancer were investigated. Intrinsic susceptibility-weighted MRI was performed before and during a period of carbogen gas breathing. Quantitative *R*_2_^*^ pixel maps were produced for each tumour and at each time point and changes in *R*_2_^*^ induced by carbogen were determined. There was a mean reduction in *R*_2_^*^ of 6.4% (*P*=0.003) for DU145 xenografts and 5.8% (*P*=0.007) for PC3 xenografts. In all, 14 human subjects were evaluable; 64% had reductions in tumour *R*_2_^*^ during carbogen inhalation with a mean reduction of 21.6% (*P*=0.0005). Decreases in prostate tumour *R*_2_^*^ in both animal models and human patients as a result of carbogen inhalation suggests the presence of significant hypoxia. The finding that carbogen gas breathing improves prostate tumour oxygenation provides a rationale for testing the radiosensitising effects of combining carbogen gas breathing with radiotherapy in prostate cancer patients.

The relationship of radiosensitivity to varying oxygenation and the detrimental effects of hypoxia in human tumours was first shown over 50 years ago (Gray *et al*, 1953; Tomlinson and Gray, 1955). Since then, it has been established that hypoxia is an important factor in radiotherapy treatment failure and has been associated in clinical studies with poor local tumour control and relapse in many cancer sites (Nordsmark *et al*, 1996; Fyles *et al*, 1998; Brizel *et al*, 1999; Movsas *et al*, 2002; Hoskin *et al*, 2003).

Eppendorf microelectrode measurements have been used to invasively characterise the range and heterogeneity of oxygen partial pressures in the prostate and show that hypoxic regions exist in human prostate carcinoma (Movsas *et al*, 1999; Parker *et al*, 2004). The outcome of radical radiotherapy for prostate cancer is influenced by the presence of hypoxia. A study that prospectively analysed 57 patients with localised disease showed that hypoxic tumours had a significantly worse biochemical relapse-free survival at 2 years (31 *vs* 92%, *P*<0.0001; Movsas *et al*, 2002). Oxygenation status is therefore an additional prognostic factor beyond the classic prognostic factors (age, clinical stage, Gleason score and prostate-specific antigen) that predicts radiation treatment failure in prostate cancer. A modelling study based on these clinical data predicts an oxygen enhancement ratio for prostate cancer of 1.4 (95% confidence interval of 1.2–1.8) that is consistent with the *in vitro* OER measurements of human tumour cell lines under chronic hypoxia conditions (Wang *et al*, 2006). These data taken together suggest that hypoxia is likely to be a valid therapeutic target in prostate cancer.

The rationale for using a high oxygen-content gas to improve tumour oxygenation is that the resulting increase in arterial pO_2_ will enhance the diffusion of soluble oxygen into tissues. Carbogen is a normobaric high oxygen content gas mixture, which is usually administered at one of the two concentrations (95% O_2_ with 5% CO_2_ or 98% O_2_ with 2% CO_2_). Carbogen has been shown to improve the oxygenation of both experimental and human tumours (Falk *et al*, 1992; Robinson *et al*, 1997; Powell *et al*, 1999; Rijpkema *et al*, 2002). This gas mixture increases intravascular oxygen availability resulting in greater oxygen uptake by tumours. It also transiently increases tumour blood flow (possibly due to a vasodilatory effect mediated by CO_2_), which increases oxygen delivery to regions of perfusion-limited hypoxia (Sibtain *et al*, 2002). Extracellular tumour pH has been shown to decline in response to carbogen gas breathing, particularly for large and hypoxic tumours (McSheehy *et al*, 1998). A study in a murine tumour model showed that the level of radiosensitisation achieved is dependent on both the CO_2_ content of the inspired gas and the duration of gas breathing (Hill *et al*, 1998).

Hypoxia modification has been shown to improve radiotherapy outcomes in several tumour sites, including the use of carbogen gas breathing in combination with hypofractionated, conventional and hyperfractionated radiotherapy schedules (Overgaard and Horsman, 1996). Despite this, there has been a paucity of investigation into its value in prostate cancer. In this study, blood oxygen level-dependent (BOLD) MRI, also called intrinsic susceptibility MRI, has been used to show the changes in blood oxygenation in response to carbogen inhalation in two animal models and in human subjects. This imaging technique capitalises on the differing magnetic properties of oxygenated and deoxygenated blood. Deoxyhaemoglobin is paramagnetic and acts as an intravascular contrast agent because it brings about an increase in the transverse relaxation rates of blood and surrounding tissues. Microscopic field gradients in the vicinity of red blood cells and vessels are modulated by the changes in the deoxyhaemoglobin concentration, which leads to signal attenuation in susceptibility-weighted MR images (Alonzi and Hoskin, 2006). Thus, the purpose of this study was to evaluate changes in prostate tumour oxygenation using BOLD MRI in response to carbogen breathing.

## Materials and methods

### Animal experiments

All animal experiments were performed in full compliance with UK government regulations and local guidelines on animal welfare and were approved by the local ethical review committee.

### Cell culture and implant

DU145 and PC3 cells were incubated under 19.6% O_2_, 5% CO_2_ and 75.4% N_2_ at 37°C. Both cell lines were originally isolated from patients with metastatic hormone refractory prostate cancer (Mickey *et al*, 1977; Stone *et al*, 1978; Kaighn *et al*, 1979). The cells were maintained in Dulbecco's Modified Eagle Media (DMEM) supplemented with 10% foetal calf serum (FCS), 2 mM L-glutamine, 100 U ml^−1^ penicillin and 100 *μ*g ml^−1^ streptomycin. Tumours were initiated by injecting (2 × 10^6^ cells) subcutaneously into the rear dorsum of 8–10-week-old SCID mice. Animals were selected for investigation when their tumours reached 8–10 mm geometric mean diameter.

### Imaging for tumour hypoxia

Mice were sedated for each MRI scan using 200 *μ*l of 1 : 10 hypnorm in water, injected into the peritoneal cavity. Sedated mice were placed in a 6-cm diameter quadrature birdcage coil (Varian, Palo Alto, CA, USA) in a 4.7 tesla Varian MR system. A hot air blower with continuous temperature monitoring was used to maintain body temperature.

Initial T_1_-weighted sagittal and transverse scans were obtained for localisation. Multiple spoiled gradient echo (GRE) images were acquired on a single central slice with increasing echo times (TE=4–48 ms, in 4 ms steps), with TR 117 ms, flip angle (*α*) 45°, slice thickness 1 mm, and field of view 40 × 45 mm. Scanning time was divided into three gas-breathing periods with mice breathing air, carbogen (95% O_2_ and 5% CO_2_) and then air again. The first air breathing period lasted 4 min, followed by 10 min of carbogen breathing and then 4 min breathing air. Images were obtained every minute, resulting in 18 images for each xenograft. All gases were administered at 1 l min^−1^ (gases obtained from BOC, UK). A vacuum-based gas scavenger was used to minimise changes in oxygen content within the magnet bore. GRE images with the same imaging parameters that were used for the baseline scan were acquired every minute throughout the experiment.

### Animal MR image analysis

Multiecho sequence images were processed using Matlab version 5 (Mathworks, Nantick, MA, USA). A region of interest (ROI) was drawn around the tumour using the initial T_1_-weighted image. *R*_2_^*^ pixel maps were then calculated for each time point of the imaging series. For each pixel, a straight line was fitted to a plot of lnS_t_ against TE for each pixel using a least-squares approach, of which the gradient is intrinsic relaxivity (−*R*_2_^*^; s^−1^).

### Statistical analysis

Repeatability was assessed by comparing two of the baseline BOLD sequences taken 1 min apart during the air-breathing period at the beginning of the carbogen experiment (time points two and three) for each xenograft. Bland and Altman (1996a, 1996b, 1996c) statistics for measuring repeatability were used. This gave a range, based on the repeatability of the entire group, of the change required in any given xenograft to be considered statistically significant at the 95% confidence level and not simply be due to the natural test variability. The effect of hyperoxia on *R*_2_^*^ was evaluated using the time series of repeated BOLD MRI scans during carbogen breathing. The first four baseline *R*_2_^*^ measurements, before the carbogen gas exposure was initiated, were averaged to give a single baseline value. The remaining *R*_2_^*^ values (measurements 5–18) were subtracted from the baseline measurement to give a *ΔR*_2_^*^ value, that is, the change in *R*_2_^*^ from baseline. Group significance testing was performed using the paired two-tailed student's *t*-test. Correlation between baseline *R*_2_^*^ and *ΔR*_2_^*^ was assessed using Pearson's correlation coefficient.

### Human testing

A total of 17 patients with prostate cancer (age=56–76 years, Gleason grade=6–8, PSA=1.9–32.0 ng ml^−1^) that were due to be treated with a radical prostatectomy procedure were recruited. None had received hormonal treatment. Investigations were performed after approval by a local institutional review board and full ethics committee approval. Written informed consent was obtained from each subject. The histological diagnosis of prostate cancer was made by core biopsy in all patients. Patients were imaged in a Symphony 1.5T MRI scanner (Siemens Medical Systems, Erlangen, Germany) using phased array pelvic coils.

### Imaging for tumour hypoxia

The small field-of-view images of T_2_-weighted anatomical scans perpendicular to the urethra were used to stage tumours and to identify tumour slice locations. Images were inspected for the presence of a peripheral zone abnormality consistent with cancer. An oncological radiologist inspected the images, and the slice location for BOLD imaging was chosen.

For each patient, five spoiled gradient-echo images were acquired for three slices through the prostate with varying TE (5–60 ms), TR=100 ms, flip angle=40°, FOV=200 mm, 256^2^ matrix, and 8 mm thickness from which *R*_2_^*^ maps were calculated. Imaging was performed at three time points: (1) initial BOLD MRI (breathing room air) to determine baseline *R*_2_^*^ values, (2) second BOLD MRI (breathing room air) performed immediately after the first BOLD scan to establish the repeatability of the BOLD technique, (3) third BOLD scan initiated after 10 min of carbogen breathing. Carbogen was administered through a tight-fitting facemask and continued for the duration of the BOLD acquisition. A 98% O_2_ and 2% CO_2_ mixture was used to maximise patient compliance (Powell *et al*, 1999).

### MRI data analysis

Tumour ROIs were outlined using the T_2_-weighted anatomical scans. In general, an irregular mass of low signal intensity seen on the T_2_-weighted images was considered to represent tumour. When an obvious malignant peripheral zone tumour was contiguous with homogeneous low signal intensity in the central gland, the ROI of interest was expanded to encompass the entire visible abnormality. A consultant radiologist with a specialist interest in prostate cancer imaging independently verified these regions (ARP).

Voxel-based calculations were performed using DiffusionView^©^ version 2.1.3, a customised analysis software package developed in IDL (Research Systems; Boulder, CO, USA) at the Institute of Cancer Research, London, UK. *R*_2_^*^ maps were created by pixel-by-pixel fitting of a straight line to a plot of lnS_t_ against TE for each pixel using a least-squares approach, of which the gradient is −*R*_2_^*^ (s^−1^). Pixels with either negative or zero values were excluded from the analysis.

### Statistical analysis

The same statistical analysis as the animal study was used for the human experiments, the only differences being that two rather than four baseline (air-breathing) time points were available from which to calculate the baseline *R*_2_^*^ measurement and only a single carbogen-breathing image was obtained.

## Results

### Animal experiments

**Figure 1:**
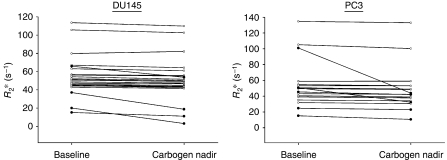
Baseline values and the effect of carbogen gas breathing on the median *R*_2_^*^ value of individual DU145 xenografts (left panel) and PC3 xenografts (right panel). Each line represents an individual tumour. Lines with filled black circles represent tumours with a change in *R*_2_^*^ that was statistically significant at the 95% confidence level and not simply a result of natural test variability. There was a mean reduction in *R*_2_^*^ of 3.52 s^−1^ or 6.4% (*P*=0.003) for the DU145 tumours and 3.04 s^−1^ or 5.8% (*P*=0.007) for the PC3 tumours.

A total of 23 DU145 tumour-bearing mice and 17 PC3 tumour-bearing mice were investigated. Baseline *R*_2_^*^ values for the two xenograft models overlapped and both showed marked heterogeneity ranging from 15.4 to 135.0 s^−1^ (Figure 1). The test proved to be extremely repeatable with a coefficient of variation (wCV) of 3.4% for DU145 tumours and 2.2% for PC3 tumours. For an individual xenograft, a reduction in *R*_2_^*^ of 9.3% for DU145 tumours and 6.3% for PC3 tumours could be considered significant at the 95% confidence level (i.e. not simply a result of test variability). Analysis on an individual tumour by tumour basis for DU145 and PC3 tumours showed that four (17.4%) of the DU145 tumour-bearing mice and five (29.4%) of the PC3 tumour-bearing mice had reductions in *R*_2_^*^ that could be considered statistically significant at the 95% confidence level (Figure 1). Analysis of the two groups as a whole showed a mean reduction in *R*_2_^*^ of 3.52 s^−1^ or 6.4% (*P*=0.003) for the DU145 tumours and 3.04 s^−1^ or 5.8% (*P*=0.007) for the PC3 tumours.

**Figure 2:**
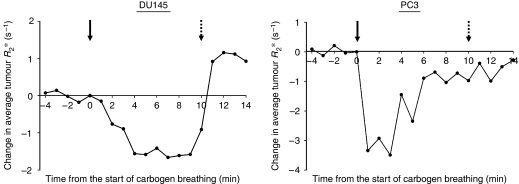
The temporal effect of carbogen breathing on the oxygenation of the 23 DU145 xenografts (left panel) and 17 PC3 xenografts (right panel). *R*_2_^*^ values fall rapidly after the commencement of carbogen breathing (arrow), as tumour oxygen levels increase. In both DU145 and PC3 tumours, *R*_2_^*^ values seem to begin a return towards their baseline reading before the carbogen exposure was terminated (dotted arrow).

The carbogen time series graphs show the temporal effect of carbogen breathing on the oxygenation of the prostate cancer xenografts (Figure 2). *R*_2_^*^ values fall rapidly after the commencement of carbogen breathing as tumour oxygen levels increase. By the end of the experiment, the *R*_2_^*^ values have essentially returned to baseline. Interestingly, in both DU145 and PC3 tumours, *R*_2_^*^ values seem to begin a return towards their baseline reading before the carbogen exposure had been terminated. For DU145 tumours, this occurs after approximately 9 min and with PC3 tumours, the effect is seen earlier, at about 4–5 min. Following the cessation of carbogen breathing, *R*_2_^*^ values for the DU145 xenografts seemed to ‘overshoot’, finishing at a higher level than at baseline. However, this difference was not statistically significant.

There was no correlation between the baseline *R*_2_^*^ value and the change in *R*_2_^*^ following carbogen exposure, for either tumour type.

### Human testing

All 17 patients completed the two baseline BOLD scans. One patient was unable to tolerate carbogen breathing, due to a feeling of claustrophobia while in the MRI scanner wearing a facemask. In two other patients, it was not possible to locate tumour on the imaging slices. This left 14 patients available for analysis.

**Figure 3:**
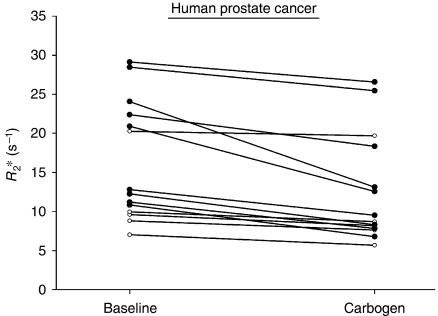
The effect of carbogen gas breathing on the median *R*_2_^*^ value of 14 individual human prostate cancers (age=56–76 years, Gleason grade=6–8, PSA 1.9–32.0 ng ml^−1^). Each line represents an individual tumour; lines with filled black circles represent tumours with a change in *R*_2_^*^ that was statistically significant at the 95% confidence level, and not simply a result of natural test variability. There was a mean reduction in *R*_2_^*^ of 3.52 s^−1^ or 21.6% (*P*=0.0005).

Baseline *R*_2_^*^ values for the human tumours were lower than those found in the xenografts because of the magnetic field strength dependence of *R*_2_^*^ relaxivities (Blockley *et al*, 2008), and also showed less heterogeneity (*R*_2_^*^=7.1–29.2 s^−1^). The susceptibility-weighted BOLD technique proved to be highly repeatable in human prostate cancer with a wCV of 4.7%. For an individual patient's prostate tumour, a change in *R*_2_^*^ of 1.95 s^−1^ (or 12.9%) could be considered significant at the 95% confidence level. Analysis of tumour regions on an individual patient-by-patient basis showed that 9 of the 14 patients (64%) had reductions in tumour *R*_2_^*^ during carbogen exposure that could be considered statistically significant at the 95% confidence level (Figure 3). Analysis of the group as a whole showed a mean reduction in *R*_2_^*^ of 3.52 s^−1^ or 21.6% (*P*=0.0005) for the prostate tumour ROIs.

As for the xenografts, there was no correlation between the baseline *R*_2_^*^ value and the change in *R*_2_^*^ following carbogen exposure.

## Discussion

This study is the first to have quantified the BOLD effect with the calculation of *R*_2_^*^ values in both prostate cancer xenografts and human tumours (these results are remarkably concordant). Using repeatability statistics, we were also able to confidently identify, on an individual basis, whether or not there is an oxygen-enhancing effect of carbogen breathing for every tumour. Two-thirds of the prostate cancer patients had a statistically significant reduction in *R*_2_^*^ following carbogen exposure. These results expand on a previous demonstration of an oxygen-enhancing effect of carbogen breathing in the benign human prostate gland (Taylor *et al*, 2001).

The two xenografts used in this study are known to be hypoxic, with median pO_2_ values shown to be 3.0–6.4 mm Hg when investigated in a study using the Eppendorf histograph (Teicher *et al*, 1994). The same report also described the heterogeneity of oxygenation measurements between xenografts that is corroborated by the wide variation in baseline *R*_2_^*^ measurements seen in the current study. The reason why there is a greater variation in baseline oxygenation in the xenografts compared with human tumours is unclear. One explanation may be that the xenografts exhibited substantial and variable degrees of necrosis with a mean of 18.1% (range=0–74.9%) of the total cross-sectional PC3 tumour area being necrotic and a mean of 9.4% (range=0–35.7%) for DU145 tumours. In contrast, necrosis is not a major feature of human prostate cancer. Areas of necrosis would have a low *R*_2_^*^ value because of the lack of viable red blood cell delivery. These regions are surrounded by an area of diffusion-limited hypoxia with a disorganised vascular network that results in a fluctuant, unpredictable blood supply, and these areas would be expected to have higher *R*_2_^*^ values.

One may expect that the most hypoxic tumours should show the greatest benefit from carbogen exposure. This has not been shown for either xenograft or for the human tumours in this study, with no correlations seen between the baseline *R*_2_^*^ value and the change in *R*_2_^*^ following carbogen exposure. This lack of concordance has been reported in earlier studies of various tumour types, using both microelectrode and imaging methods for hypoxia detection (Teicher *et al*, 1994; Howe *et al*, 1999; Powell *et al*, 1999; Taylor *et al*, 2001). It would appear that it is not necessarily the most hypoxic tumours that exhibit the greatest response from carbogen breathing. However, as defined by R_2_^*^, tumours that demonstrate a clear response are hypoxic. Also, it seems that separate areas of equivalent hypoxia within a single tumour may not respond equally to carbogen stimulation. These findings reflect the complexity and heterogeneity of the microregional vascular and endocrine environment within tumours. As a result, it is only possible to use BOLD-MRI to measure the response to carbogen breathing, but not to predict which tumours are likely to respond to carbogen exposure.

The use of BOLD-MRI for assessment of tissue hypoxia is based on the assumption that oxygenation of haemoglobin is related to blood arterial pO_2_ (as described by the oxygen-haemoglobin dissociation curve), which is in equilibrium with oxygenation of surrounding tissues. Several studies have shown that *R*_2_^*^ changes in response to vasomodulation with carbogen are temporally correlated with changes in tissue pO_2_ (Al-Hallaq *et al*, 1998; Robinson *et al*, 1999; Taylor *et al*, 2001). Tumours appear to differ in their ability to respond to carbogen inhalation with only 50–60% of human tumours showing changes in *R*_2_^*^ (Taylor *et al*, 2001). The reasons for even hypoxic tumours having limited and heterogeneous responses are complex, but undoubtedly include the fact that tumours may have adapted to widely different perfusion environments. It is to be noted that even when vessels are present, red blood cell transport through these vessels may not be effective (Robinson *et al*, 2003). Thus, hypoxic tumours with high blood volume (due to high microvessel density coupled with large vessels) will have raised baseline *R*_2_^*^ values. On the other hand, hypoxic tumours with low or zero blood volume, due to necrosis, lower microvessel density or due to small vessels, will have lower baseline *R*_2_^*^ values because of the lack of delivery of red blood cells to tissues. Attempts have been made to combine blood volume information with *R*_2_^*^ maps to improve the sensitivity and specificity for hypoxia detection (Alonzi *et al*, 2008). Once robust protocols are available, more accurate estimation of the oxygen-enhancing effect of carbogen breathing in prostate cancer may be possible.

Limitations of the BOLD-MRI technique used here are: (1) it does not measure tissue pO_2_ directly (either in blood or in tissues because of a non-linear relationship of *R*_2_^*^ and tissue pO_2_); (2) images obtained have low signal to noise ratio; (3) the current implementation is single slice, but multislice sequences are now available. The primary advantages of BOLD-MRI are there is no need to administer exogenous radioactive contrast material, and images at high temporal and with high spatial resolution can be obtained and repeated as needed; it is possible to decouple the effects of flow and deoxyhaemoglobin, which are intrinsic to native BOLD images, and so to show the changes in oxygenation independent of changes in the blood flow.

The series of experiments reported in this study show that prostate cancer is amenable to hypoxia modification using carbogen gas breathing. Prostate tumour *R*_2_^*^ decreases by an average of 5.8–6.4% in animal models and by 21.6% in human patients as a result of carbogen exposure. These results support the clinical testing of the radiosensitising effect of combining carbogen gas breathing with radiotherapy in prostate cancer.
